# The school environment and asthma in childhood

**DOI:** 10.1186/s40733-015-0010-6

**Published:** 2015-10-08

**Authors:** Marissa Hauptman, Wanda Phipatanakul

**Affiliations:** 1 Division of General Pediatrics, Boston Children’s Hospital, Boston, MA USA; 2 Division of Allergy and Immunology, Boston Children’s Hospital, Boston, MA USA; 3 Harvard Medical School, Boston, MA USA; 4 Region 1 New England Pediatric Environmental Health Specialty Unit, Boston, MA USA; 5 Boston Children’s Hospital, 300 Longwood Ave., Boston, MA 02115 USA

**Keywords:** School exposures, School-based environmental intervention, Pediatric asthma, School-based asthma management

## Abstract

In this article, we discuss the relationship between environmental exposures within the school environment and pediatric asthma morbidity. This article will conclude by reviewing novel school based asthma education and therapeutic programs and environmental interventions designed to help mitigate pediatric asthma morbidity.

## Background

Asthma is the most common childhood disease, affecting up to 15 % of children in the United States (U.S.) [1, 2]. The burden of asthma is not distributed evenly with urban minority children of low socioeconomic status enduring higher morbidity [3]. In addition to health care utilization, in 2013, pediatric asthma was the leading cause of school absenteeism and accounted for an annual loss of more than 10.5 million school days per year [4]. Data from the U.S. National Interview Survey found that children with asthma missed 3 times more school days and had a 1.7 times increased risk of suffering from a learning disability as compared to children without asthma [3].

It has been well studied that aeroallergen, mold, and airborne pollutant exposure in the inner-city home environment is associated with significant childhood asthma morbidity [5–10]. While the home environment has been extensively studied, the U.S. school environment is less well understood, largely due to the logistical and community hurdles. Despite this, numerous U.S. based and European studies have demonstrated considerable allergen and pollutant levels present in the inner-city school environment, where children spend 7–12 hours per day, which may be contributing to asthma morbidity [11–26]. Additionally, increases in asthma exacerbations and hospitalization have been observed among children 2–3 weeks after return-to-school following holidays, especially summer holidays [27, 28].

In this article, we discuss the relationship between environmental exposures within the school environment and pediatric asthma morbidity. This article will conclude by reviewing novel school based asthma education and therapeutic programs and environmental interventions designed to help mitigate pediatric asthma morbidity. We have focused on inner-city school environments due to the disproportionately high asthma burden in these areas [5–10]*.* Although, the primary disease of interest in this review article is childhood asthma, environmental exposures and interventions within the school environment may impact morbidity of other allergic and irritant-induced diseases such as eczema and allergic rhinitis [29].

## Review

### School environmental exposures: allergens

The school environment is a significant reservoir for allergens, pollutants, and viral respiratory infections [11–26]. In 2009, a comprehensive review of allergen exposures in schools highlights the routine exposure to variable levels of indoor allergens in schools dependent on building characteristics, geographic, climatic and cultural factors [11]. As in home environments, it is unlikely that a single school or classroom based environmental exposure is exclusively responsible for asthma morbidity [30, 31].

The school environment may be an important site of exposure to indoor allergens, including cockroach, cat, dog, mouse, dust-mite, and molds, known to be important in the urban home environment [32]. Higher asthma morbidity in inner-city children has historically been associated with cockroach and mouse allergens more than other commonly encountered allergens in home environments [10, 33–35]. Previous studies found cockroach and mouse allergens highly prevalent in school environments [13, 21]. The ***S***chool ***I***nner-***C***ity ***A***sthma ***S***tudy (SICAS) is a NIH/NIAID funded, comprehensive, prospective study of inner-city school and classroom specific exposures and asthma morbidity among inner-city students in the Northeast [36]. In SICAS, our study group has reliably detected much higher levels of mouse allergen in schools, compared to the same students’ home environments [22, 24], with levels similar to those seen in occupational lab animal settings [37]. Cat and dog allergen levels in the school environment in SICAS were variable [22], and not at levels previously shown to worsen symptoms [38]. European school-based studies have demonstrated cat and dog allergens at high levels in schools, likely from passive transfer of students who owned pets in their homes [20, 32]. Consistent with other studies, there was very little cockroach allergen discovered [22, 32]. Dust mite allergen in schools and day care facilities are found in similar or slightly lower levels than in corresponding respective homes, and given their propensity to thrive in humid environments, highest average concentrations were detected in humid regions in the United States and Brazil [11, 39]. Some of the differences between the European and U.S. inner-city cohorts are likely due to climatic, cultural and occupant factors [32].

### School environmental exposures: mold

Schools are a unique microenvironment of indoor air pollutants and particulates, as well as associated mold and other allergens carried on these particles. An ongoing prospective study evaluating indoor air pollution in Europe, entitled The Health Effects of Indoor Air Pollutants (HITEA), has found high levels of mold in schools, particularly those with moisture damage [29, 40–43]. These mold findings substantiate the results from SICAS, which found elevated levels of mold in settled dust and airborne concentrations [25]. This was further substantiated by a recent national Taiwanese study, which demonstrated that fungal spore levels in classrooms correlated with asthma symptoms and a relief of symptoms on weekends and holidays [44].

### School environmental exposures: near roadway proximity and indoor air quality

Schools are typically centrally located within a community and a recent study conducted by Kingsley et al. demonstrated that approximately 3.2 million (6.5 %) children across the United States attended schools located within 100 meters of a major roadway as defined by the United States Census Bureau [45]. In addition, to being in close proximity to heavy traffic routes and commercial or industrial exposures, schools frequently serve as a hub for pick-up, drop-off, and idling of cars and buses, potentially contributing to a site-specific increase in ambient pollution that are not characterized by typical definitions of major roadways or traffic density [46].

Annessi-Maesano et al. [47], as part of the French 6 Cities Study, assessed indoor air quality data in primary schools and investigated the relationships between classroom based air pollutants and asthma and rhinitis in schoolchildren, this study, however, did not comprehensively adjust for home environmental mold and allergen exposure levels. This study demonstrated that overall about one-third of the 6,590 schoolchildren were exposed to high concentrations of air pollutants as defined by the World Health Organization for fine particulate matter with aerodynamic diameter ≤ 2.5 μm (PM2.5) and nitrogen dioxide (NO_2_), levels above 10 μg/m3 and 40 μg/m3, respectively [47]. In multivariate linear mixed regression models, asthma was more common in classrooms with high PM2.5, after adjusting for age, gender, passive smoking, maternal or paternal history of asthma, dampness, gas appliance, ethnicity and socio-economic status [47]. When the population was stratified by skin prick test positivity, significant positive associations were identified among PM2.5 and NO_2_ and sensitized asthmatics. Other international studies conducted in urban areas of Taiyuan, China [48] and Barcelona, Spain [49], corroborated these findings.

Further exacerbating indoor air quality, classroom activity re-suspends indoor air particles thereby increasing exposure [50]. Children are frequently physically active in school, increasing their minute ventilation and thus the inhaled dose of pollutant concentrations [50]. Schools also sometimes have poor ventilation [51] and suffer inadequate building maintenance [52]. A review study conducted by Daisy et al. [53], found that classroom ventilation is typically inadequate and may exacerbate children’s exposure to indoor air pollutants. This review article, highlighted a study conducted by Smedje et al. [54, 55], which showed that 41 % of carbon dioxide measurements in 38 schools located in the fourth largest city of Sweden were above 1000 parts per million (ppm), the threshold generally regarded as indicative of unacceptable ventilation rates.

### School-based asthma management programs

Several national, state, and city governmental and non-governmental organizations including the American Lung Association [56], Allergy and Asthma Foundation of America [57], National Heart, Blood and Lung Institute(NHLBI), the Centers for Disease Control and Prevention’s (CDC) National Asthma Control Program, which includes 36 state and territorial state asthma programs [58], and the Environmental Protection Agency’s Indoor Air Quality Tools for Schools Program have developed a number of school-based asthma programs. These major school-based activities include school-based asthma therapeutic management programs, self-management education for students, indoor air quality and trigger reduction programs, educational trainings for school personnel and administering asthma medication self-carry law [59]. State asthma programs utilize the data from their CDC-funded asthma surveillance systems to focus activities in regions with the most hospitalizations and emergency department visits for asthma. These multidisciplinary programs work with state asthma partnerships to identify areas with high health risk students and to identify evidence-based interventions to implement statewide [58].

A review study published in 2011, demonstrates that school based asthma education programs that teach self-management, knowledge, and skills to children and adolescents with asthma, are effective in decreasing school absenteeism related to asthma with less definitive findings on reduced health care utilization metrics up to the first year post-interventions [3, 60–68]. An example of school based asthma education programs with ongoing success is the American Lung Association’s Open Airways for Schools, which is implemented throughout the United States [62, 69]. It has been sustained through use of undergraduate-level health education students [62, 69] and similar programs have demonstrated success with medical students in Australia [70].

A randomized control trial conducted by Noyes et al. [71, 72], which assessed the effectiveness of administration of a daily dose of preventive asthma medication within the school setting was effective and cost-effective in reducing symptoms in inner-city children with asthma as compared to usual care [64]. This may be especially important for inner-city pediatric populations where inhaled corticosteroids are especially underused, with median usage rates of only 32 % among African-Americans compared with 51 % among Caucasians [73]. Several small studies of supervised daily control therapy at school have corroborated this randomized control trial with improvements in adherence and health outcomes [3, 74–76]. There have been documented success, in settings where a consulting physician worked with school nurses, resulting in increases in albuterol treatments at school and subsequent reductions in students being sent home or requiring emergency services for further treatment [3, 77]. Lastly, a larger randomized controlled trial showed marginally significant improvements among students new to controller therapy when treated at school compared to home [3, 78].

Bruzzesse et al., comprehensive review of school based asthma programs, highlighted competing priorities in the education system, which present challenges to the implementation of school-based asthma programs. Among these challenges, is the importance of a school nurse in the success of these management programs. National Association of School Nurses, documents that only 45 % of schools had a full time registered nurse or licensed practical nurse [59]. Limited studies [61, 67, 79–81] with mixed results suggest that there is a potential for an innovative intervention targeting school-based personnel beyond school nurses or school based health care settings and further studies are needed to determine their effectiveness.

### School-based environmental interventions

A perspective published in 2014 [29], highlighted the limited nature of school-based environmental intervention studies done to date and proposed feasible school-based environmental interventions to mitigate asthma morbidity. Prior school-based environmental intervention studies have been small, cross-sectional, and did not uniformly control for exposures in the home environment [12–16, 18, 19, 21, 26, 29, 82–86]. To fully understand if school-based environmental interventions improve asthma morbidity, investigators must also collect information on the home environment.

Several small longitudinal studies in Europe have found improvement in asthma symptoms with repair of air filtration systems, repair of moisture damage, and reduction in mold exposure and other building maintenance [55, 87, 88]. A small-randomized trial in Australia found that when controlling for the home environment, replacing school heaters and thus reducing NO_2_ levels reduced asthma symptoms [29, 83]. Although effective in controlling particle concentrations, these types of heaters are not routinely used in schools in the United States, and most schools do not utilize gas stoves, making indoor sources of NO_2_ less likely [29]. A study conducted by Beatty et al., assessed the health impact and cost effectiveness of a new localized emissions reduction program that retrofits diesel school buses with aggressive pollution control technologies in the State of Washington [89]. This study was associated with statistically significant and large reductions in respiratory illness incidence among at-risk children and adults with chronic respiratory conditions within the greater Puget Sound region, which includes Seattle [89].

In Sweden, interventions to reduce pet dander in schools have been conducted, although such interventions—including pet avoidance measures or even banning pet ownership—would not be practical in the United States [84–86]. Additionally, given the low level of cat and dog dander found in prior inner city school-based studies, this may not be an effective intervention to reduce asthma exacerbations within the U.S. inner city pediatric populations [22].

Given the scarcity of comprehensive data on school-based environmental interventions and health outcomes, successful home-based strategies currently serve as the model for school-based interventions [29]. A landmark study by Morgan et al., from the Inner-City Asthma Study Group showed that multifaceted removal of multiple allergens and pollutants through allergen-impermeable covers, HEPA filter vacuum cleaners, HEPA air purifiers, and professional pest control could improve asthma outcomes [90]. One potential school-based intervention is the use of air filtration systems to reduce environmental exposures [91, 92]. A recent report on air filtration outlined what is known in this field and called for more rigorous trials and research [91]. With regard to types of air filtration systems, room HEPA air filters may be more practical for study purposes [91, 93], and may be utilized to control classroom-specific exposures. If successful within single classrooms, these results may inform future school-wide policies and practices.

Similarly, a pilot study showed that HEPA filters reduce mold spore counts in daycare centers, which have similar conditions to a school environment [94]. Another example of a feasible school-based environmental intervention is integrated pest management. Given the markedly high levels of mouse allergen in schools compared to levels in children’s individual bedrooms [22, 23], our group piloted strategies toward comprehensive effective school-based environmental reduction techniques and tailored components, such as integrated pest management. These environmental controls were modeled from successful home-based strategies and adapted for tolerance and acceptability in a school and classroom, to collectively reduce allergen and pollutant levels in preparation for a NIH/NIAID funded School Inner-City Asthma Intervention Study [29].

Despite the logistical challenges of implementing comprehensive school-based environmental, educational and therapeutic interventions, evidence provides support towards the contribution of school and classroom exposures and health outcomes [55, 83–88, 94]. School-based interventions have the potential to reduce exposures for many symptomatic children, in contrast to the individual families impacted by home-based interventions. If effective, results from school-based interventional studies could inform public policy change, funding, and initiatives [29]. While establishment and implementation of public policies is an expensive undertaking for cities, preliminary studies suggest that environmental interventions may be cost beneficial [95]. In inner cities where the burden of disease is so great, interventions may reduce the cost to the community even further.

## Conclusions

The school environment where children and school personnel spend a majority of their day is a significant reservoir for allergens and pollutants [11–26]. There are several domains to which to intervene on school based asthma surveillance, education, optimization of asthma management and adherence to recommendations as well as environmental interventions that all have the potential to mitigate pediatric asthma morbidity. If it can be demonstrated that reduction of classroom-specific exposures and other therapeutic and educational interventions lead to improved asthma outcomes, then findings can be translated into cost-effective strategies to benefit communities of children through improvement of the school environment. In this limited resource environment, it will be critical to determine, which are most efficient and cost-effective to implement broadly to improve pediatric asthma morbidity.

## Abbreviations

CDC: Centers for Disease Control and Prevention
HEPA: High-efficiency particulate arrestance
HITEA: Health effects of indoor air pollutants
NIH: National Institutes of Health
NIAID: National Institute of Allergy and Infectious Disease
NHLBI: National Heart, Lung, and Blood Institute
NO2: Nitrogen dioxide
PM_2.5_: Particulate matter with diameter ≤ 2.5 μm
ppm: Parts per million
SICAS: School Inner-City Asthma Study
U.S.: United States
